# Adsorption Isotherm and Mechanism of Ca^2+^ Binding to Polyelectrolyte

**DOI:** 10.1021/acs.langmuir.3c03640

**Published:** 2024-03-18

**Authors:** Sriteja Mantha, Alec Glisman, Decai Yu, Eric P. Wasserman, Scott Backer, Zhen-Gang Wang

**Affiliations:** † Division of Chemistry and Chemical Engineering, California Institute of Technology, Pasadena, California 91125, United States; ‡ Division of Chemistry and Chemical Engineering, California Institute of Technology, Pasadena, California 91125, United States; § Core R&D, The Dow Chemical Company, 633 Washington St., Midland, Michigan 48674, United States; # Consumer Solutions R&D, The Dow Chemical Company, 400 Arcola Road, Collegeville, Pennsylvania 19426, United States

## Abstract

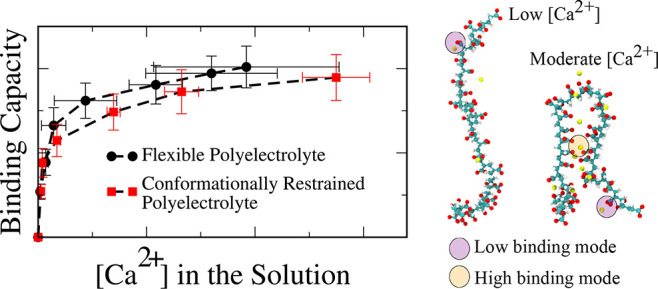

Polyelectrolytes, such as poly(acrylic acid) (PAA), can effectively mitigate CaCO_3_ scale formation. Despite their success as antiscalants, the underlying mechanism of binding of Ca^2+^ to polyelectrolyte chains remains unresolved. Through all-atom molecular dynamics simulations, we constructed an adsorption isotherm of Ca^2+^ binding to sodium polyacrylate (NaPAA) and investigated the associated binding mechanism. We find that the number of calcium ions adsorbed [Ca^2+^]_ads_ to the polymer saturates at moderately high concentrations of free calcium ions [Ca^2+^]_aq_ in the solution. This saturation value is intricately connected with the binding modes accessible to Ca^2+^ ions when they bind to the polyelectrolyte chain. We identify two dominant binding modes: the first involves binding to at most two carboxylate oxygens on a polyacrylate chain, and the second, termed the high binding mode, involves binding to four or more carboxylate oxygens. As the concentration of free calcium ions [Ca^2+^]_aq_ increases from low to moderate levels, the polyelectrolyte chain undergoes a conformational transition from an extended coil to a hairpin-like structure, enhancing the accessibility to the high binding mode. At moderate concentrations of [Ca^2+^]_aq_, the high binding mode accounts for at least one-third of all binding events. The chain’s conformational change and its consequent access to the high binding mode are found to increase the overall Ca^2+^ ion binding capacity of the polyelectrolyte chain.

## 1 Introduction

Divalent metal ions, such as Ca^2+^, exhibit a pronounced affinity for dissolved anions like carbonate (CO_3_^2–^) in an aqueous solution. The ion pairs, which readily associate, precipitate out of solution and form solid deposits (scale) due to their low solubility limit. Scale formation presents challenges to residential and industrial piping systems, restricting the fluid flow and fouling components.^1−3^ These metal ions also form complexes with household products such as detergents and disrupt their efficacy.

Polyelectrolytes, such as poly(acrylic acid) (PAA), are commonly employed to mitigate scale formation.^4−8^ Although it is not fully understood what makes an effective antiscalant polyelectrolyte, a few mechanisms for scale prevention have been proposed. The polyelectrolytes could prevent nucleation via chelating metal ions from the solution.^5,9^ The chelation reduces the concentration of the metal ion and the likelihood of their association with the dissolved anions. Simultaneously, polyelectrolytes could also adsorb onto the surfaces of scale crystals and prevent further growth or deposition.^6,7,10,11^

The solution behavior of polyelectrolytes in divalent salt solutions poses important challenges in designing polyelectrolytes with enhanced antiscalant activity, as the polyelectrolyte–ion complex itself can precipitate and lead to further scale deposition.^12−23^ Cloud point measurements revealed instances of phase separation into a polymer-poor (supernatant) liquid and a polymer-rich liquid with addition of divalent ions.^24^ Boisvert et al. performed osmotic pressure measurements and showed that the solution behavior of polyelectrolytes in divalent salt solutions is primarily influenced by a proposed site-binding mechanism of divalent cations.^25^ The site-binding mechanism facilitates bridging of nonadjacent repeat units by the divalent cations. Through a Fourier transform infrared (FTIR) dialysis technique,^26^ Fantinel et al. identified monodentate, bidentate, and bridging modes when Ca^2+^ ions bind to carboxylate groups of a polyacrylate chain. However, the relative importance of each of these binding modes—particularly that of the bridging mode—on the ability of polyelectrolyte to chelate Ca^2+^ ions has not been elucidated. Furthermore, the influence of the Ca^2+^ ion concentration on these binding modes remains elusive.

Mean-field theories have shed light on the solution behavior of polyelectrolytes in divalent salt solutions.^27−34^ In addition to establishing conditions for the precipitation behaviors, these theoretical models have predicted a large reduction in polymer size with addition of divalent ions,^35^ beyond what is expected from electrostatic screening. The chain collapse was primarily attributed to ion bridging between non-neighboring repeat units. Coarse-grained implicit solvent molecular simulations, employing generic models for polyelectrolyte chains and ions, have confirmed the chain collapse and attributed it to the bridging capability of divalent ions.^35−37^ All-atom molecular dynamics simulations have gone a step further by explicitly treating solvent molecules to investigate the molecular principles that govern the binding of divalent cations to the polyelectrolyte chain.^38−44^ These investigations have each reported that the Ca^2+^ ion is strongly coordinated with the polyelectrolyte chain, resulting in highly coiled conformations with a chain rigidity reminiscent of crystal-like structures. Because of the strong Ca^2+^–polyelectrolyte interactions and the number of Ca^2+^ ions binding to the polyelectrolyte chain, these models hint at the overcharging of the Ca^2+^–polyelectrolyte complex. However, recent potentiometric titration assays^9^ suggest that only one-third of the binding sites are occupied before a fully charged polyelectrolyte chain reaches its saturated value of Ca^2+^-binding capacity.

The saturation value in the adsorption isotherm, which describes the maximum amount of Ca^2+^ that can be bound to a polyelectrolyte chain, reflects the chelating capacity of the polyelectrolyte chain. However, the molecular principles that govern the corresponding adsorption isotherms have not been addressed. Specifically, the interplay between the site-binding nature of Ca^2+^–polyelectrolyte interactions and the conformational transitions of the polyelectrolyte, aimed at enhancing both the chelating capacity and the solubility of the Ca^2+^–polyelectrolyte complex, has not been explored.

In this study, we address the mechanism of Ca^2+^ adsorption onto a polyacrylate chain. We constructed an adsorption isotherm to describe the binding behaviors of Ca^2+^ ions to polyacrylate. We then determined the different binding modes accessible for binding of Ca^2+^ to the chain and quantified their impact on the adsorption isotherm. In our follow-up paper,^45^ we address questions related to Ca^2+^ ion-mediated association between polyelectrolyte chains. The rest of the paper is organized as follows. In Section 2, we present a Hamiltonian replica exchange molecular dynamics (HREMD) protocol to selectively bias Ca^2+^–polyelectrolyte interactions and efficiently sample the configurational space. We then introduce a free energy perturbation approach coupled with molecular dynamics to compute the adsorption isotherm describing binding of Ca^2+^ to the polyelectrolyte chain. We discuss the results of these calculations in Section 3 and present the conclusions in Section 4.

## 2 Methods

We investigated the mechanism of binding of calcium ions to polyacrylate chains using all-atom molecular dynamics (MD) simulations. Our simulation system consists of a single poly(acrylic acid) (PAA) chain with 32 repeat units, solvated in a cubic water box with an edge length of 12 nm. All repeat units on the polymer are charged, consistent with the solution conditions for antiscalant activity (i.e., solution pH ∼10). Sodium ions were added for electroneutrality. The average end-to-end distance of such a polymer was ∼5 nm. We chose box dimensions so that the polymer does not interact with the periodic image. In Table 1, we report compositions of different systems studied in this work.

**Table 1:** Composition of the Cubic Simulation Box with an Edge Length of 12 nm, Containing a Single Sodium Polyacrylate Chain with 32 Repeat Units, and at Various Numbers of CaCl_2_ in Water

label	Ca^2+^	Cl^–^	Na^+^	water
0CaCl_2_	0	0	32	56448
4CaCl_2_	4	8	32	56436
8CaCl_2_	8	16	32	56424
16CaCl_2_	16	32	32	56400
32CaCl_2_	32	64	32	56352
64CaCl_2_	64	128	32	56256
96CaCl_2_	96	192	32	56160
128CaCl_2_	128	256	32	56064

### Force Field Choice and the Importance of Solvent Electronic Polarization

We employed the generalized AMBER force field (GAFF) with the SPC/E water model, which had been rigorously tested by Mintis et al. for modeling the polyacrylate chain.^46^ During our modeling of calcium ions, we observed that the “full” electrostatic charge force field parameters overestimated calcium ion binding to carboxylate groups on the polyacrylate chain, resulting in a charge inversion of the polymer chain inconsistent with experimental reports (Figure S2). Duboué-Dijon et al., who investigated calcium ion binding to insulin, reported that such an overestimation was due to inadequate treatment of electronic dielectric screening when using full charges on ions with nonpolarizable force fields.^47^ The correct energetics of ion-pair formation could be captured by molecular models with explicit polarization.^48^ However, a general-purpose force field with explicit polarization, tested to reproduce the properties of polyacrylates, was not readily available, and polarizable force fields introduce a large computational expense.

Alternatively, in our models, we included electronic dielectric screening by uniformly scaling the charges of all of the solute atoms. This approach, known as the electronic continuum charge correction (ECC), is a mean-field method that attempts to mimic charge-carrying species within an electronic dielectric continuum.^49,50^ While the ECC scheme is a physically meaningful concept, it is primarily used as an *ad hoc* solution to incorporate electronic polarization into an otherwise nonpolarizable force field. ECC schemes tend to fail in systems with a discontinuity in the high-frequency dielectric constant.^51^ Moreover, force field parametrizations implicitly account for electronic polarization effects to some extent. Introducing additional ECC correction may overly compensate for electronic polarization effects, resulting in a significant underestimation of cohesive energy density and leading to unphysical solution behavior.^52^ Nevertheless, the ECC scheme yields meaningful observations in common electrolyte systems where the high-frequency dielectric constant remains uniform throughout.

Within the scope of our study, the interactions of interest involve calcium ions and carboxylate groups on the polyacrylate chain. Biomolecular systems with the same carboxylate–calcium ion pair have shown success with ECC schemes.^53−56^ We particularly employ the ECC scheme reported by Jungwirth and co-workers, who provided parameters for modeling calcium and other ions in an aqueous solution.^54^ In their work, the authors uniformly scaled the charges on ions by a factor of 0.75. Lennard-Jones parameters describing dispersion interactions of corresponding ions were optimized to reproduce *ab initio* molecular dynamics results for ion-pairing and neutron scattering experiments. Such a parameter set accurately described the properties of calcium ions in an aqueous solution and their association with carboxylate groups on amino acids. We used the same parameter set to model calcium and other ions in our simulation system. Although the ECC scheme is tailored to the specific requirements of modeling the polyacrylate–Ca^2+^system of interest in this work, the underlying rationale—addressing electronic polarization to correct for electrostatic sources of overestimated binding affinities—is a principle that governs modeling a broad class of polyelectrolyte systems.

When modeling the electronic polarization effects of the polyacrylate chain in an aqueous solution, we scaled the partial charges of all the atoms on the polyacrylate chain by 0.75 and used Lennard-Jones parameters reported by Mintis et al.^46^ to describe dispersion interactions. Although our approach is a commonly accepted practice,^56^ we emphasize that it is not rigorous. However, different properties of polymers strongly depend on their chain length, and finding the right strategy to optimize their dispersion interaction parameters is not obvious.

Because the “full-charge” parameters by Mintis et al.^46^ reproduce the structural properties of the polyacrylate chain in a salt-free solution, we validated the predictions of the “scaled-charge” model against the former. Even though we did not reoptimize the Lennard-Jones interaction parameters of the polyelectrolyte chain, the conformational flexibility of a polyelectrolyte chain in a salt-free solution did not change. Additionally, we observed identical distributions for structural properties when compared to the full-charge” model (see Figure S3).

### Simulation Methodology

We used GROMACS 2022.3^57−59^ patched with PLUMED 2.8.1^60−62^ and employed the following protocol to conduct our molecular dynamics simulations of the systems reported in Table 1. First, we minimized the energy of the system using the steepest descent algorithm until the maximum force on any atom in the system was smaller than 100 kJ mol^–1^ nm^–1^. Next, we equilibrated the system at constant NPT conditions with the pressure and temperature set to 1 atm and 300 K, respectively. While fluctuations in the energy and box size minimized within a few nanoseconds of the equilibration run, it took tens of nanoseconds to equilibrate the chain structure. We used the Berendsen barostat^63^ with the velocity-rescaling stochastic thermostat during the first 10 ns of the equilibration. For the remainder of the equilibration run, we switched to the Parrinello–Rahman barostat^64^ with the Nosé–Hoover chain thermostat for the accurate reproduction of the thermodynamic properties of the system.^65,66^ Following the equilibration run, we conducted production MD simulations of these systems in the NVT ensemble using a Nosé–Hoover chain thermostat.

We employed the leapfrog time integration algorithm with a finite time step of 2 fs to integrate the equations of motion. Additionally, we utilized the LINCS constraint algorithm to convert all bonds with hydrogen atoms into constraints.^67^ We applied periodic boundary conditions along all three spatial axes and used the particle mesh Ewald (PME) method with a minimum Fourier spacing of 0.12 nm to calculate the long-ranged electrostatic interactions.^68,69^ We applied a cutoff distance of 1.2 nm for computing van der Waals interactions. We used the same distance as the real-space cutoff value while computing the PME electrostatics.

### Need for Enhanced Sampling Molecular Simulations

Although the ECC scheme with the nonpolarizable force field greatly reduced the PAA–Ca^2+^ binding/unbinding relaxation times, regular MD simulations were unable to efficiently sample the polymer conformational space in an aqueous CaCl_2_ solution. Even a microsecond long trajectory was not sufficient to sample polymer conformations in any of the systems listed in Table 1 with calcium numbers higher than 4 Ca ions. We direct readers to Figures S4 and S5 for the relevant data and discussion.

To address the challenges associated with polymer conformational sampling in an aqueous CaCl_2_ solution, we employed Hamiltonian replica exchange molecular dynamics (HREMD).^70,71^ Our HREMD framework is based on the flexible implementation of the REST2 variant,^70^ as previously reported by Bussi.^71^ We introduced a parameter λ to selectively bias the interactions between the polymer and ions as well as the dihedral potential components of the Hamiltonian. The charges of the ions and the polymer were scaled by a factor of , while their Lennard-Jones interaction parameter (ϵ) was scaled by λ. Similarly, the polymer dihedral potential was also scaled by λ. With this scheme for λ-parametrized Hamiltonian, λ = 1 corresponds to the system of interest with full-scale interactions. We determined that the parametrized Hamiltonian with λ = 0.67 rapidly sampled the polymer conformational space. Coordinate exchange between neighboring replicas was attempted every 500 steps. We utilized 16 replicas, with λ values ranging from 1 to 0.67 (geometrically spaced), to simulate polymer conformations in an aqueous solution containing 8 CaCl_2_ or 16 CaCl_2_. For higher Ca^2+^ numbers, we increased the number of replicas to 24. This combination of the number of replicas and the λ-range yielded acceptable exchange probabilities (∼0.3) between neighboring replicas. All the relevant results reported in the subsequent sections were obtained by averaging over a 250 ns production HREMD run, conducted at constant volume and a temperature of 300 K. Our specific choices of λ-parametrization and the number of replicas in the HREMD setup were driven by the unique challenges posed by the polyacrylate–Ca^2+^ system due to long ion pair relaxation and resulting inefficiencies in sampling polymer backbone conformations. Nevertheless, the conceptual approach of employing replica exchange variants to overcome sampling bottlenecks in polymer conformational sampling is broadly applicable to other polyelectrolyte–multivalent ion complexes.^18^

### Computing Ion Adsorption Isotherm from Molecular Dynamics Simulations

The primary objective of this work is to investigate Ca^2+^ chelation onto a model polyacrylate chain. In an aqueous CaCl_2_ solution containing a polyacrylate chain, a dynamic equilibrium exists between calcium ions that are freely dispersed in the solution (Ca_aq.free_^2+^) and the calcium ions adsorbed per monomer of the polyacrylate chain (AA–Ca^2+^).
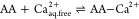
1Here, AA represents the concentration of repeat units on the polymer chain that are not bound to any calcium ions. An adsorption isotherm, quantifying eq 1, describes the calcium ion chelating ability of a model polyacrylate chain. We constructed the isotherm by plotting the number of calcium ions adsorbed per monomer (AA–Ca^2+^) against the concentration of calcium ions that are freely dispersed in the solution (Ca_aq.free_^2+^).

Computing AA–Ca^2+^ from the simulation trajectory is straightforward. We calculated the number of calcium ions within 0.7 nm (Supporting Information) of the polymer atoms at each frame. This quantity was then divided by the number of repeat units per chain (i.e., 32 in this case) and ensemble averaged over the trajectory.

We determined Ca_aq.free_^2+^ by equating the chemical potential of the Ca^2+^ ions in the system (μ_CaCl_2__^System^) with that of a pure CaCl_2_ aqueous suspension (μ_CaCl_2__^Solution^). This required conducting simulations of two separate sets of systems: one containing a polyacrylate chain to determine μ_CaCl_2__^System^ and the other without a polyacrylate chain for μ_CaCl_2__^Solution^. We employed the procedure laid out by Panagiotopoulos and co-workers to determine the chemical potentials via free energy perturbation approach.^72−75^

In brief, μ_CaCl_2__ represents the change in free energy when adding (or removing) a unit of CaCl_2_. This is expressed in eq 2 as the sum of the corresponding ideal gas component (μ_CaCl_2__^id^) and the residual component (μ_CaCl_2__^R^).
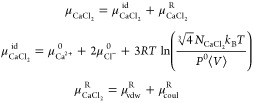
2Here, μ_Cl^–^_^0^ and μ_Ca^2+^_^0^ are the respective chemical potentials of Cl^–^ and Ca^2+^ at a reference pressure of 1 bar. We used the tabulated value of μ_Cl^–^_^0^ from the NIST-JANAF thermochemical tables.^76^ Moučka et al. estimated the value for μ_Ca^2+^_^0^,^77^ and we employed the same value in our calculations. N_CaCl_2__ represents the number of CaCl_2_ units in the simulation box, *k*_B_ denotes the Boltzmann constant, *P*^0^ = 1 bar is the reference pressure, and ⟨*V*⟩ stands for the average volume obtained from NPT simulations of a system with a specific composition.

To calculate μ_CaCl_2__^R^, we gradually decoupled Ca^2+^ and two Cl^–^ from the system. The initial configuration for this study came from the most probable polymer conformation identified from the previously described enhanced sampling simulations. We then tagged a Ca^2+^ ion that was adsorbed onto the polymer backbone. In a solution without the polyacrylate chain, a randomly selected Ca^2+^ ion served as the tagged ion and a comparison point between the two systems. Regardless of the system, two randomly chosen Cl^–^ ions were tagged.

The electrostatic interactions of the tagged Ca^2+^ ion with other particles in the system were gradually turned off in 11 stages (ϕ = 1, 0.9, ..., 0). Here, ϕ = 1 represents the system with fully activated electrostatic interactions of the tagged calcium ion, while ϕ = 0 corresponds to complete deactivation. Each stage consisted of a series of molecular dynamics simulation steps, beginning with energy minimization, followed by 5 ns of equilibration and a 10 ns production simulation run at a pressure of 1 bar and a temperature of 300 K. The output from the production step of the *i*th stage (ϕ_*i*_) served as the initial configuration for the MD simulation steps of the (*i* + 1)th stage (ϕ_*i*+1_). The same methodology was then applied to deactivate the electrostatic interactions of each tagged chloride ion and also the van der Waals interactions of the tagged calcium ion and the two tagged chloride ions. We then used the Bennett acceptance ratio (BAR)^78,79^ approach, natively implemented in the GROMACS MD package,^57^ to estimate the residual chemical potential (μ_CaCl_2__^R^) from these different stages of MD simulations. Notably, given the position-dependent nature of the unbinding free energy of a Ca^2+^ ion from the polymer backbone, we calculated the unbinding free energy for each adsorbed Ca^2+^ ion individually and used their average to estimate the μ_CaCl_2__^R^.

## 3 Results and Discussion

**Figure 1:**
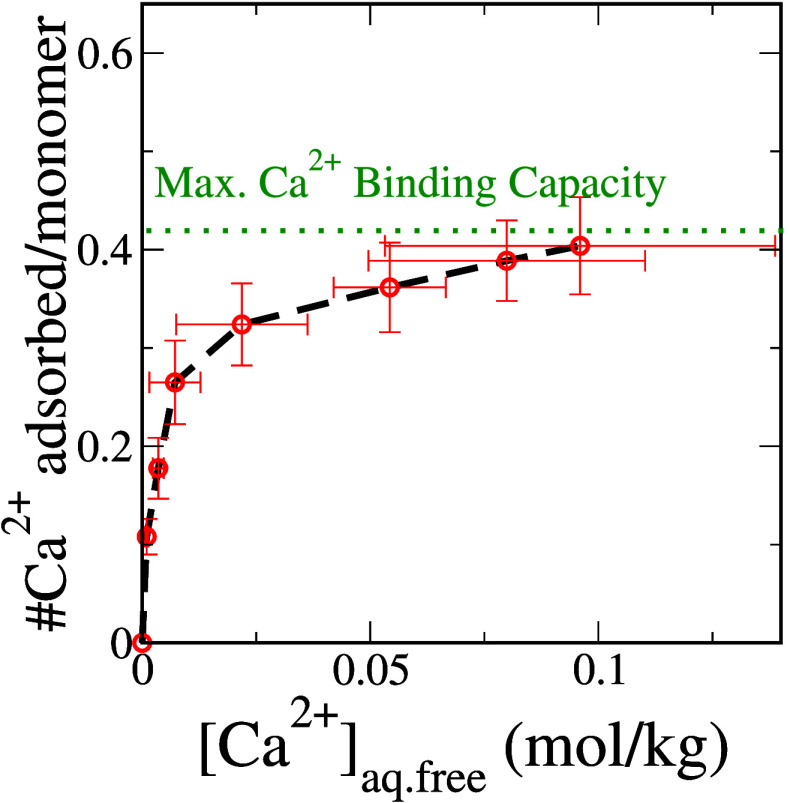
Isotherm describing Ca^2+^ ion binding to a polyacrylate chain with 32 repeat units. Error bars represent one standard deviation around the sample mean. Note: the apparent positive slope of the last two data points is a consequence of large uncertainties in determining the corresponding solution concentration of free Ca^2+^ in the system. The differences in their vertical axis values are very minimal (see Figure S8 and the accompanying discussion in the Supporting Information).

The ability of a polyacrylate chain to sequester Ca^2+^ ions is intricately tied to the bulk solution concentration of Ca^2+^, denoted as [Ca^2+^]_aq.free_. In Figure 1, we present the adsorption isotherm and describe the extent of binding of Ca^2+^ onto a 32-mer polyacrylate chain as a function of [Ca^2+^]_aq.free_. At low Ca^2+^ concentrations, most of the binding sites on the polyacrylate chain are available, and an increase in [Ca^2+^]_aq.free_ results in a rapid increase in the number of Ca^2+^ ions sequestered by the polyacrylate chain. However, at moderate concentrations, the accessible binding sites become occupied, and the polyacrylate chain saturates with Ca^2+^.

From the plateau in the adsorption isotherm, we note that the maximum binding capacity of a model polyacrylate chain is 0.40 ± 0.05 Ca^2+^ ions per monomer, which aligns with recent potentiometric titration experiments.^9^ Although the adsorption isotherm bears some resemblance to a Langmuir model, we observe that Ca^2+^ binding does not obey the model assumptions. Notably, the conformational flexibility of the polyelectrolyte chain results in distinct chemical environments around each binding site, rendering the binding sites nonequivalent.

**Figure 2:**
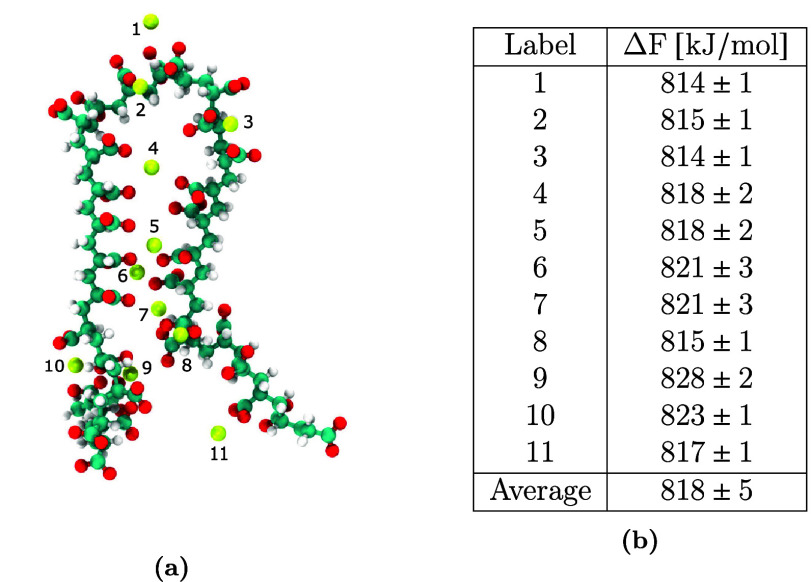
(a) Conformation of a 32-mer polyacrylate chain with the most probable radius of gyration in a solution corresponding to [Ca^2+^]_aq.free_ = 0.026 mol/kg and (b) free energies of unbinding a Ca^2+^ ion that is adsorbed on the polyacrylate chain.

We demonstrate this phenomenon in a polyelectrolyte solution corresponding to Ca_aq.free_^2+^ = 0.026 mol/kg. First, we identify the system configuration that corresponds to the polymer chain with the most probable radius of gyration (Figure 2a). Then, we independently unbind each of the bound Ca^2+^ ions. We employ the free energy perturbation approach described in Section 2 to compute the unbinding free energy and report the corresponding values in Figure 2b.

The unbinding free energies of the 11 Ca^2+^ ions broadly fall into two classes. We categorize these binding sites into a “low” binding mode (≤818 kJ/mol) and “high” binding mode (>818 kJ/mol). From the binding sites depicted in Figure 2a along with the accompanying free energies in Figure 2b, we identify that the high binding mode is approximately 5–10 kJ/mol energetically more favorable than the low binding mode and facilitates a Ca^2+^ ion bridge between non-neighboring carboxylate groups on the polyacrylate chain.

**Figure 3:**
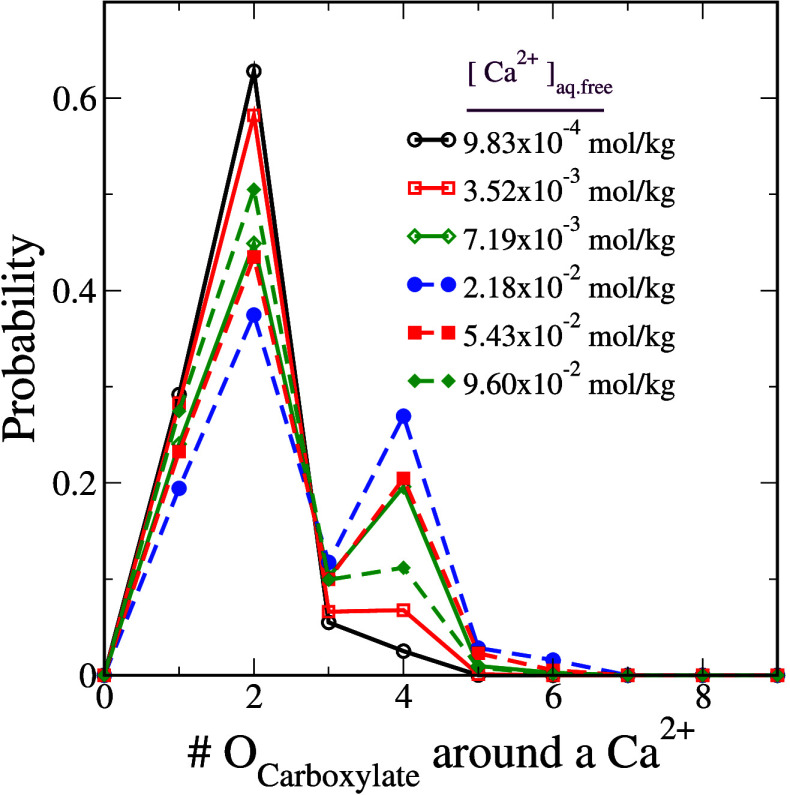
Probability of finding a certain number of carboxylate oxygen atoms (O_Carboxylate_) on a 32-mer polyacrylate chain around a Ca^2+^ ion.

The various binding modes and their coupling to the polyelectrolyte conformation could impact the number of Ca^2+^ ions sequestered. We determine a binding mode by tracking the number of carboxylate oxygen atoms around a Ca^2+^ ion. From the radial distribution of carboxylate oxygen atoms around a Ca^2+^ ion, we identify that their most probable separation is about 0.35 nm (see the Supporting Information). We compute the probability of finding a varying number of carboxylate oxygen atoms within 0.35 nm from a Ca^2+^ ion and report this as a function of Ca_aq.free_^2+^ in Figure 3.

We observe that the low binding mode, in which two carboxylate oxygen atoms bind to a given Ca^2+^ ion, remains dominant at all concentrations of Ca_aq.free_^2+^ studied. However, with an increase in Ca_aq.free_^2+^, the high binding mode corresponding to ion bridging becomes more favorable. Interestingly, when Ca_aq.free_^2+^ exceeds 2.18 × 10^–2^ mol/kg, this trend reverses: further increases in Ca_aq.free_^2+^ promote the low binding mode once more. We hypothesize that this nonmonotonic trend in the population of different binding modes arises from the enhanced electrostatic screening at higher Ca^2+^ concentrations.

**Figure 4:**
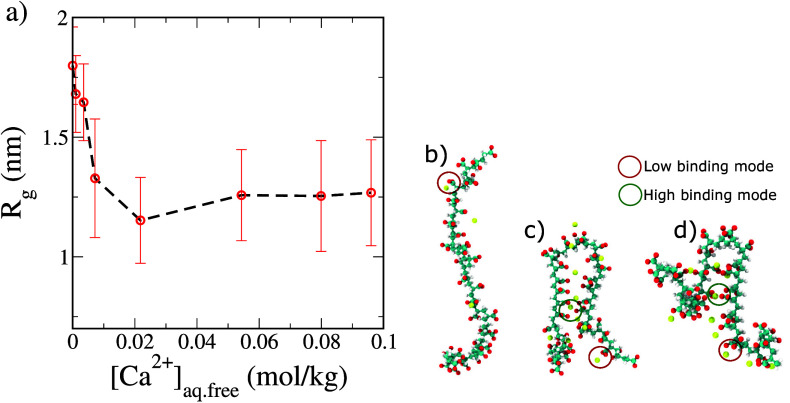
(a) Radius of gyration of 32-mer polyacrylate chain at different concentrations of Ca_aq.free_^2+^. The large values of standard deviation indicate chain conformational flexibility. Panels b–d illustrate the dominant binding modes at low, moderate, and high Ca_aq.free_^2+^ concentrations, respectively. The conformation in panel c corresponds to the *R*_g_ minimum in panel a, indicating a hairpin-like compact state of the polyacrylate chain at moderate calcium concentrations.

Nevertheless, at moderate and high concentrations of Ca_aq.free_^2+^, nearly one-third of the binding events are due to the high binding mode. This high coordination binding environment is seen to have a large effect on the polymer size and conformation. We investigate the coupling between the binding modes and the polyelectrolyte chain conformation by tracking the chain radius of gyration (*R*_g_) as a function of Ca_aq.free_^2+^. We report these results in Figure 4.

We note from Figure 4a that at low concentrations of Ca_aq.free_^2+^, where the population of the high binding mode is insignificant, the polymer chain adopts an extended conformation with an *R*_g_ of approximately 1.65–1.9 nm (Figure 4b). At these concentrations, Ca^2+^ ions bind to at most one carboxylate group on the polyacrylate chain. As Ca_aq.free_^2+^ increases and the population of high coordination binding sites subsequently increases, the polymer conformation transitions to a hairpin-like state (Figure 4c). Here, the high coordination binding sites, which bridge two strands of the polyelectrolyte chain, are nearly as prominent as the low coordination binding sites located on the solvent-exposed side of each strand. Intriguingly, in solutions with high concentrations of Ca^2+^ ions, although some of the bridging events are disrupted, the conformation of a polyelectrolyte chain still resembles that of a hairpin-like state (Figure 4d). The disruption of the bridging events due to enhanced screening from the other ions in the system increases the conformational flexibility and hence the average chain size.

**Figure 5:**
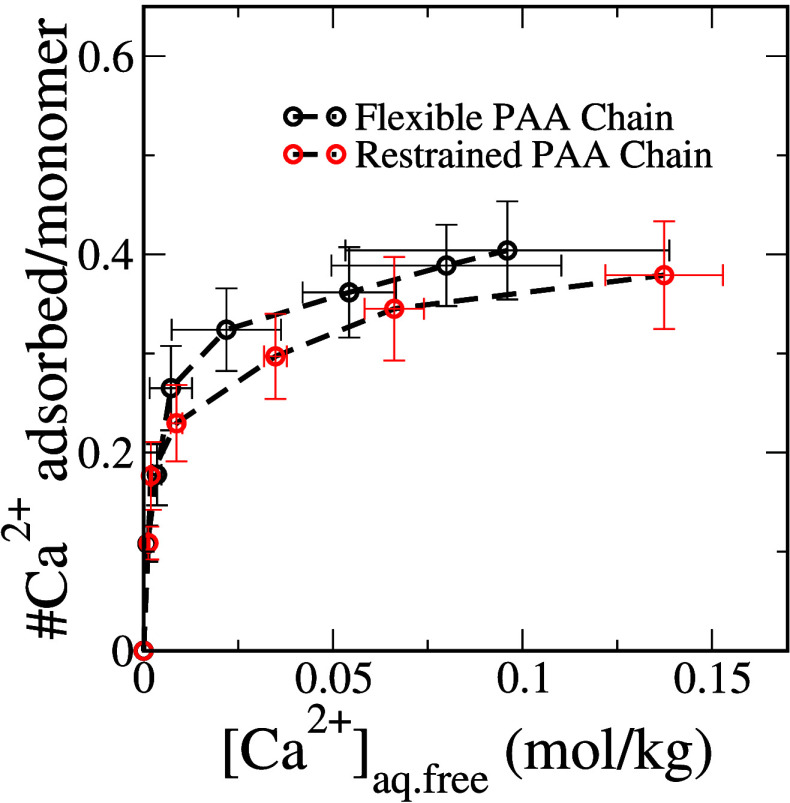
Ca^2+^ ion binding isotherm to a polyacrylate chain with 32 repeat units restrained to an extended coil conformation and unrestricted.

Because the high coordination binding sites are energetically more favorable, we anticipate that access to a larger population of these binding sites would enhance the ability of a polyelectrolyte chain to sequester Ca^2+^ ions. To explore this, we investigate the inverse problem; we limit the polyelectrolyte chain to only binding sites with low coordination environments and construct the adsorption isotherm. We achieve this by first identifying the polymer conformation that corresponds to the most probable *R*_g_ in a system with no added Ca^2+^ ions. Utilizing this chain conformation, with harmonic restraints imposed, we prepared polyelectrolyte solutions with added Ca^2+^ ions. We computed the Ca^2+^ adsorption isotherm from these systems and compared it to that obtained from unrestrained simulations reported earlier. We show these results in Figure 5.

We find that at low concentrations the number of Ca^2+^ ions adsorbed on the polyacrylate chain in both the restrained and unrestrained simulations is indistinguishable. This is because at lower concentrations of Ca^2+^ ions in the solution, unrestrained polyelectrolyte chains can access only the low coordination binding sites. However, we see a noticeable difference at moderate and high concentrations, where the unrestrained polyacrylate chain favors the high coordination binding sites. The restrained simulations, which do not have access to high binding modes, consistently adsorb fewer Ca^2+^ ions per chain compared to the unrestrained simulations.

These observations indicate that a polyelectrolyte chain’s ability to efficiently chelate Ca^2+^ ions is impacted by its access to a large number of high coordination binding sites. These sites are energetically more favorable for ion binding, and as such, they provide a more stable environment for the ions, leading to more effective sequestration. This insight could prove useful in applications where selective and efficient ion capture is paramount, such as in water treatment processes or biomedical applications.

## 4 Conclusion

Using all-atom molecular simulations coupled with a free energy perturbation approach, we constructed an adsorption isotherm to describe the binding of Ca^2+^ ions to a model polyacrylate chain. Analysis of the adsorption isotherm revealed that the per-monomer Ca^2+^ ion binding capacity of a fully charged polyelectrolyte chain saturates at a value of 0.40 ± 0.05. This saturation value correlates with the binding modes accessible to Ca^2+^ ions, as they bind to the polyelectrolyte chain. Two predominant binding modes were identified: one mode involves Ca^2+^ ions binding to at most two carboxylate oxygen atoms on a polyacrylate chain, and the other involves Ca^2+^ ions binding to four or more carboxylate oxygen atoms.

The population of low binding mode sites remains high across all concentrations of Ca^2+^ in the solution. Nevertheless, at least one-third of the binding events at moderate and high concentrations of Ca^2+^ ions in the solution are defined by high binding mode events. These binding events, while responsible for enhancing the Ca^2+^ ion binding capacity of a polyelectrolyte chain, also lead to the collapse of the polyelectrolyte chain’s conformation to a hairpin-like state. The solution concentration of Ca^2+^ ions corresponding to the conformational transition falls within the range close to the supernatant (polymer-poor) side of the phase behavior reported by Sabbagh et al.^24^ The adsorption isotherm constructed in this study suggests that at these concentrations, the hairpin-like polyelectrolyte chain had attained the saturation value of the Ca^2+^ binding capacity.

The collapse of a polyelectrolyte chain into a hairpin-like conformation, with all available binding sites saturated, may indicate the onset of putative phase separation. This observation carries important implications for the chelating capacity of a polyelectrolyte toward Ca^2+^ ions and, consequently, for its antiscalant activity. Yet, a polyelectrolyte–divalent ion complex falling out of equilibrium is inherently a multichain problem. In our follow-up work,^45^ we investigate the importance of different binding modes of Ca^2+^, identified in the current work, on the association between like-charged polyelectrolyes and establish the conditions under which a polyelectrolyte in a divalent salt solution falls out of equilibrium.

## Supporting Information Available

Overcharging of the polyacrylate–Ca^2+^ complex when using nonpolarizable force fields with full charges on ions; validation of scaled charge force fields for modeling polyacrylate conformations; evidence for meaningful sampling of polyelectrolyte–Ca^2+^ complexes in an aqueous solution using a nonpolarizable force field with electronic continuum correction and Hamiltonian replica-exchange molecular simulations; chemical potential equivalence conditions and concentration of free Ca^2+^ ions in the solution that is in equilibrium with the system containing the polyacrylate chain (PDF)
