# Supplementary material for: Adsorption Isotherm and Mechanism of Ca2+ Binding to Polyelectrolyte
Source: Langmuir. 2024 Mar 18;40(12):6212–9. doi: 10.1021/acs.langmuir.3c03640 (PMC10976897; doi:10.1021/acs.langmuir.3c03640)
Supplement: Supplementary file 1 — la3c03640_si_001.pdf [file la3c03640_si_001.pdf]

Supporting Information: Adsorption isotherm and mechanism of Ca^{2+} binding to polyelectrolyte

Sriteja Mantha,[†,‖] Alec Glisman,[‡,‖] Decai Yu,[¶] Eric P. Wasserman,[§] Scott Backer,[§] and Zhen-Gang Wang[*,†]

†*Division of Chemistry and Chemical Engineering, California Institute of Technology, Pasadena, California 91125, USA*

‡*Division of Chemistry and Chemical Engineering, California Institute of Technology, Pasadena, California 91125,USA*

¶*The Dow Chemical Company, Core R&D, 633 Washington St., Midland, Michigan 48674, USA*

§*The Dow Chemical Company, Consumer Solutions R&D, 400 Arcola Road, Collegeville, Pennsylvania 19426, USA*

‖*Contributed equally to this work*

E-mail: zgw@caltech.edu

Contents

S1 Electronic continuum corrected partial atomic charges of a polyacrylate chain **S-3**

S2 Overcharging of Polyelectrolyte—ion Complex in Models with Full Ion Charges **S-3**

S3 Validating Scaled Charge Forcefield for modeling Polyelectrolyte Conformations **S-5**

S4 Meaningful Sampling with Electronic Continuum Correction and Hamiltonian Replica-Exchange Molecular Simulations **S-6**

S5 Determining $\left[\mathrm{Ca}^{2+}\right]_{\mathrm{aq.free}}$ from the equivalence of $\mu_{\mathrm{CaCl_2}}^{\mathrm{system}}$ and $\mu_{\mathrm{CaCl_2}}^{\mathrm{solution}}$ **S-7**

S6 Role of water in Ca^{2+} binding to polyacrylate chain **S-8**

S7 Additional notes on Ca^{2+} adsorption to polyacrylate chain **S-9**

References **S-10**

S1 Electronic continuum corrected partial atomic charges of a polyacrylate chain

Our molecular model for the polyacrylate chain is based on the GAFF force field parameters as reported by Mintis et al.[S1]. In order to account for polarization effects, we apply electronic continuum correction by scaling only the partial charges found in the Mintis et al. report by 0.75. As commonly practiced, we leave the corresponding Lennard-Jones interaction parameters untouched.

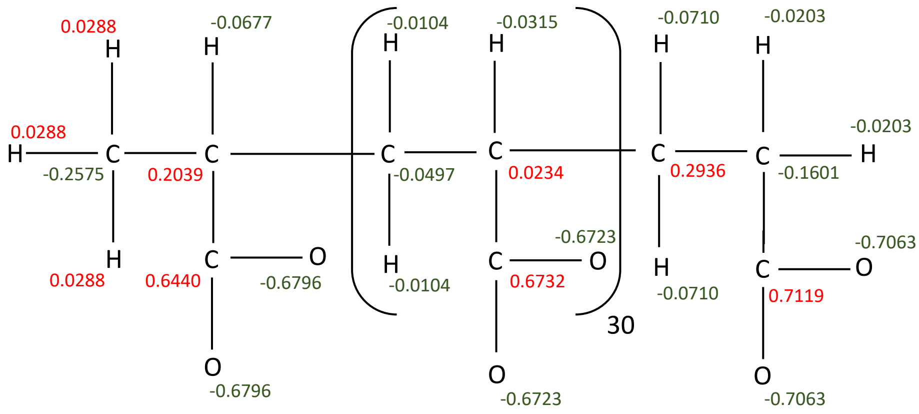

Figure S1. Scaled partial charges on all the atoms in a 32-mer polyacrylate.

S2 Overcharging of Polyelectrolyte—ion Complex in Models with Full Ion Charges

Molecular dynamics simulations, which utilize non-polarizable force fields with "full charge" models for ions, tend to overestimate the binding of dissolved ions to the polyelectrolyte chain. We demonstrate this in Figure S2(a) for a fully charged polyacrylate with 32 monomers per chain and sodium ions added to balance the charge on the polymer, in an aqueous $CaCl_2$

solution. In the figure, we report the net charge of the polyacrylate-ion complex as a function of the number of Ca^{2+} ions in the system. From the radial distribution of Ca^{2+} ions around a backbone carbon on the polyacrylate chain (see Figure S2(b)), we identify that the Ca^{2+} ions are most likely to be found at a separation of 0.7 nm from the polymer carbon. We identify all the ions (Ca^{2+}, Na^{+}, Cl^{-}) that are located within 0.7 nm from the polymer backbone and label them as condensed ions. The net charge of the polyacrylate-ion complex is calculated by summing the partial charges of the condensed ions.

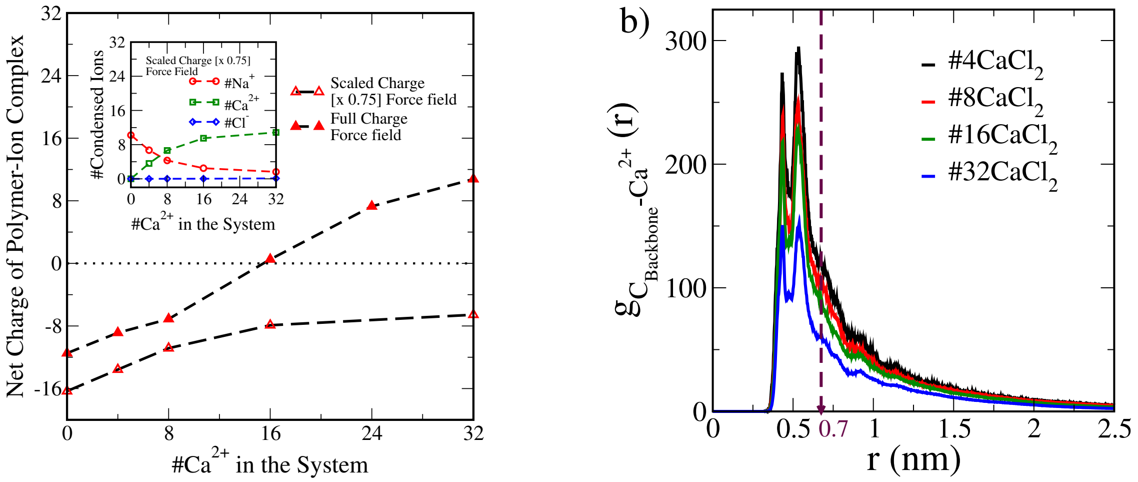

Figure S2. a) Net Charge of a Polyelectrolyte-Condensed Ion Complex: A Comparison between Results from a Full Charge and Scaled Charge Force Field. b) Radial Distribution of Ca^{2+} Ions around a Backbone Carbon on the Polyacrylate Chain Computed from Simulations Using a Scaled Charge Force Field.

We observe from Figure S2(a) that simulations with the "full charge" model yield a shift in the net charge of the polyacrylate-ion complex from negative to positive with an increase in Ca^{2+} concentration in the system. In contrast, the "scaled charge" models described in Section 2 predict an increase in the net charge, which eventually plateaus at a negative value for higher Ca^{2+} concentrations. This observation from the "scaled charge" model aligns with recent potentiometric titrations conducted by Gindele et al.[S2].

S3 Validating Scaled Charge Forcefield for modeling Polyelectrolyte Conformations

It is not clear beforehand whether the scaled charge-corrected polyacrylate chain spans the desired conformational space. Mintis et al.[S1] rigorously tested their full charge models against experimentally determined properties of polyacrylate chains in salt-free solutions. We hypothesize that if the scaled charge model for the polyacrylate chain spans the same conformational space, then it will not significantly alter their structural properties. We confirm this observation through the data presented in Figure S3.

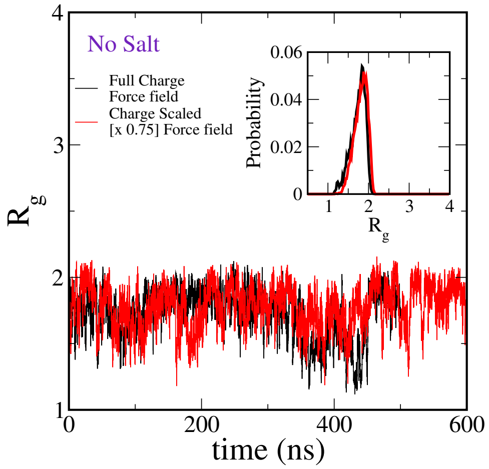

Figure S3. Temporal variation of the radius of gyration(R_g of a polyacrylate chain in an aqueous solution without any added salt

In the figure, we present the temporal variation of the radius of gyration (R_g) of the polyacrylate chain in a salt-free aqueous solution. We observe that the range of R_g values covered by both the full charge models and the scaled charge models are very similar. This observation is further supported by the R_g probability distribution shown in the inset of the figure.

S4 Meaningful Sampling with Electronic Continuum Correction and Hamiltonian Replica-Exchange Molecular Simulations

Charge scaling has significantly improved the binding/unbinding relaxation times of polyacrylate-Ca^{2+} interactions. Figure S4(a) illustrates the decay of the ion-pair survival probability autocorrelation function[S3] to zero within the time scales achievable in molecular simulations using modern GPU architecture. However, a trajectory spanning 500 ns proved inadequate for sampling the conformational space with statistical certainty. The temporal variation of R_g, as reported in Figure S4(b), indicates that the conformational space explored by polyelectrolyte chains in an aqueous solution with a concentration equal to or greater than $8CaCl_2$ is not representative of an equilibrium distribution.

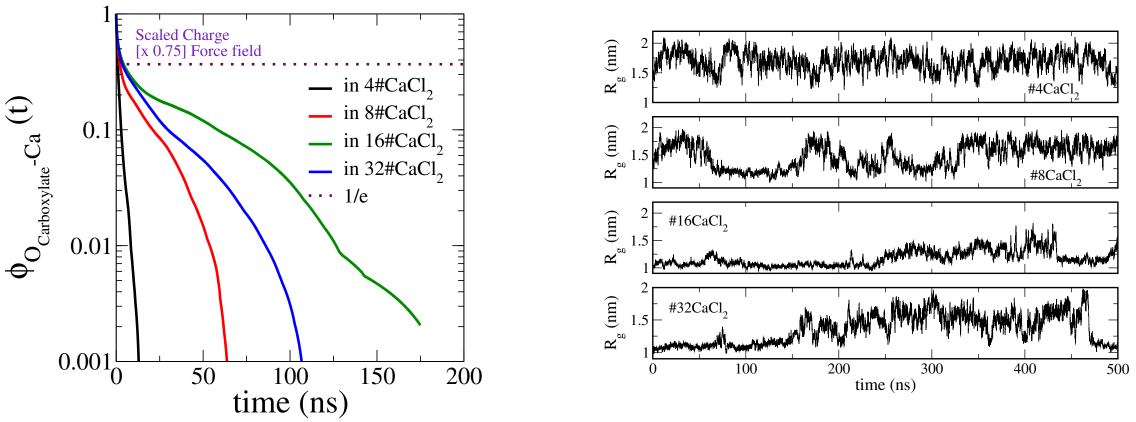

Figure S4. a) Binding/unbinding relaxation of a carboxylate oxygen –Ca^{2+} ion pair with the scaled charge force field. The function $\phi(t)$ represents the autocorrelation function depicting ion-pair survival probability[S3]. b) Temporal variation of the radius of gyration (R_g) of a polyacrylate chain in an aqueous solution with varying concentrations of $CaCl_2$.

Estimating the required length of a simulation trajectory to generate an equilibrium distribution is challenging. To overcome this issue, we use a Hamiltonian replica Exchange protocol described in Section 2 that specifically biases polyacrylate-Ion interactions in the system. In Figure S5, we present the temporal evolution of the radius of gyration of a fully charged PAA-32mer chain at different Ca^{2+} concentrations in the system, computed from

the HREMD simulation. A 100 ns trajectory already demonstrates that the simulations have explored many possible chain conformations under given system conditions.

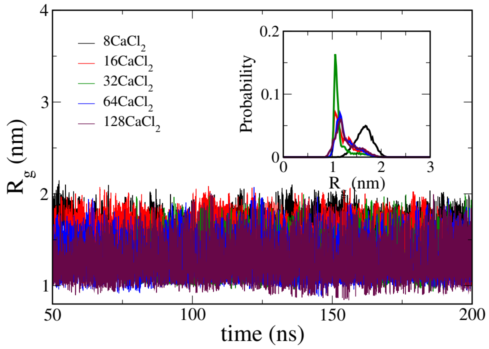

Figure S5. Predictions for the radius of gyration (R_g) of a polyacrylate chain in an aqueous solution with varying concentrations of $CaCl_2$, computed using Hamiltonian Replica Exchange simulations with a scaled charge force field.

S5 Determining $\left[Ca^{2+}\right]_{aq.free}$ from the equivalence of $\mu^{system}_{CaCl_2}$ and $\mu^{solution}_{CaCl_2}$

$\left[Ca^{2+}\right]_{aq.free}$ represents the concentration of free Ca^{2+} ions in the solution that are in equilibrium with those adsorbed on a polyacrylate chain. We determined $\left[Ca^{2+}\right]_{aq.free}$ by equating the chemical potential of $CaCl_2$ in the system with the polyacrylate chain ($\mu^{system}_{CaCl_2}$) to the chemical potential without the polyacrylate chain ($\mu^{solution}_{CaCl_2}$). We report these chemical potentials in Figure S6 and establish their equivalence conditions.

We note that the lowest $\left[CaCl_2\right]_{aq}$, without polyacrylate chain, we simulated in this work is 0.013 mol/kg. As practised by Pangiotopolous et al[S4], we assumed this concentration of $\left[CaCl_2\right]_{aq} = 0.013$mol/kg to be sufficiently low for Debye-Huckle limiting law for electrolyte solutions to hold true. For any concentrations of $\left[CaCl_2\right]_{aq} < 0.013$mol/kg, we used Debye-Huckle limiting law to compute $\mu^{solution}_{CaCl_2}$.

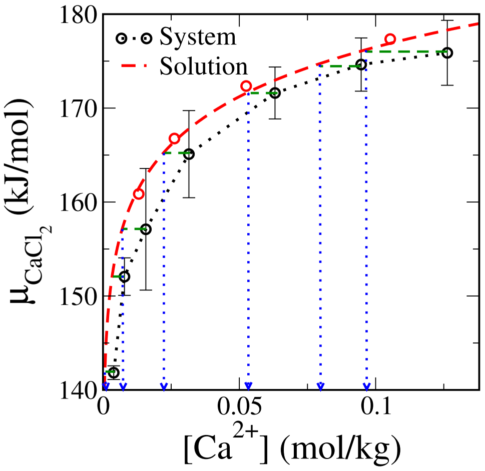

Figure S6. The chemical potential of $CaCl_2$ is computed from both the system with the polyacrylate chain and the solution without the polyacrylate chain. The horizontal green dashed lines indicate the equivalence of the $CaCl_2$ chemical potential between the System and the Solution. The vertical blue dotted lines indicate the concentration of free calcium ions in the solution that are in equilibrium with the system of interest.

S6 Role of water in Ca^{2+} binding to polyacrylate chain

Multivalent ions such as Ca^{2+} bear a strong solvation shell. When a Ca^{2+} ion, freely dispersed in the solution, approaches the polyacrylate chain to bind with the carboxylate oxygen on the polymer backbone, a restructuring of the solvation shell is anticipated to facilitate Ca^{2+} binding to the polymer chain.

We find that Ca^{2+} ions bind directly to the carboxylate oxygen, without mediation by water molecules. This is evidenced by the strong first peak in the radial distribution of Ca^{2+} around a carboxylate oxygen located at 0.25 nm (see fig. S7(a)). Intriguingly, a free Ca^{2+} sheds about two water molecules as it approaches the polyacrylate chain and binds to a carboxylate oxygen. As illustrated in the fig. S7(b), such a phenomenon is invariant to the concentration of Ca^{2+} in the solution. While the corresponding exchange dynamics would facilitate a deeper understanding of the adsorption/desorption of Ca^{2+} from the polymer chain, the HREMD method in our study obscures any real-time dynamics.

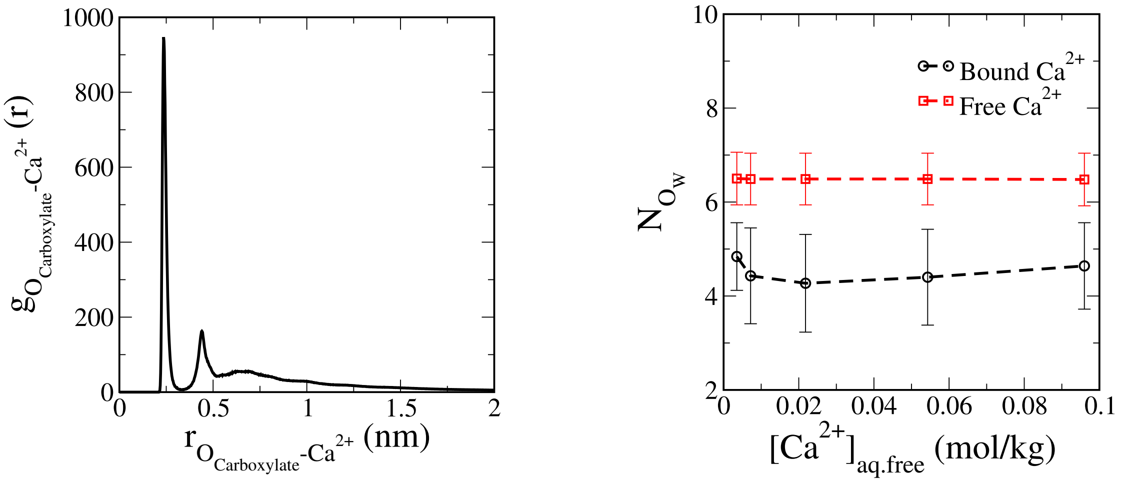

Figure S7. a) Radial distribution of Ca^{2+} around a carboxylate oxygen ($O_{Carboxylate}$) on a PAA-32mer in an aqueous solution with $[Ca^{2+}]_{aq.free} = 2.18 \times 10^{-2}$ mol/kg. b) Number of water oxygens (N_{O_W}) within the first solvation shell of Ca^{2+}. A bound Ca^{2+} ion is defined as one that is bound to any of the carboxylate oxygens. A free Ca^{2+} ion is one that is not bound to a carboxylate and is freely dispersed in the solution.

S7 Additional notes on Ca^{2+} adsorption to polyacrylate chain

The number of Ca^{2+} ions adsorbed on the PAA chain reaches a saturation value within an error margin at moderate to high concentrations of Ca^{2+} in the system. This becomes evident when we plot the number of Ca^{2+} ions adsorbed on the polyacrylate chain as a function of the number of Ca^{2+} ions added to the system, as demonstrated in fig. S8. The same observation

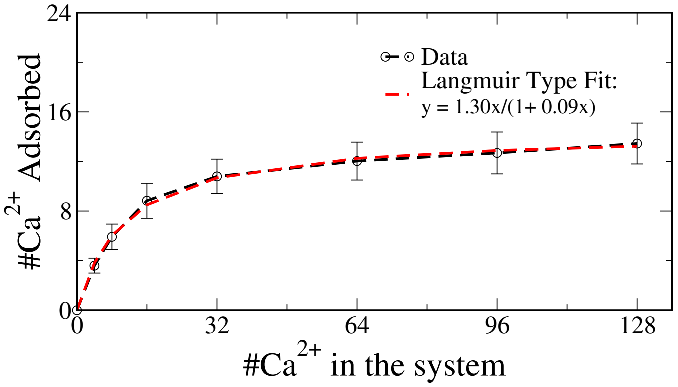

Figure S8. Number of Ca^{2+} ions adsorbed on a PAA-32mer as a function of the number of Ca^{2+} ions added to the simulation system. (Note: The fit to the Langmuir model is included only to serve as a visual aid for the saturation in the number of Ca2+ ions adsorbed on a PAA-32mer. It is not intended to indicate the adsorption mechanism.)

may not be apparent in Figure 1, where we plotted the number of Ca2+ ions adsorbed per monomer as a function of the solution concentration of Ca^{2+}, due to uncertainties in determining the latter. These uncertainties likely reflect the different binding environments that are accessible for Ca2+ binding to the polyelectrolyte chain (and hence their chemical potential), particularly at moderate to high concentrations of Ca2+ ions in the solution.

References

(S1) Mintis, D. G.; Mavrantzas, V. G. Effect of pH and Molecular Length on the Structure and Dynamics of Short Poly(acrylic acid) in Dilute Solution: Detailed Molecular Dynamics Study. *The Journal of Physical Chemistry B* **2019**, *123*, 4204–4219, DOI: 10.1021/acs.jpcb.9b01696.

(S2) Gindele, M. B.; Malaszuk, K. K.; Peter, C.; Gebauer, D. On the Binding Mechanisms of Calcium Ions to Polycarboxylates: Effects of Molecular Weight, Side Chain, and Backbone Chemistry. *Langmuir* **2022**, *38*, 14409–14421, DOI: 10.1021/acs.langmuir.2c01662.

(S3) Impey, R. W.; Madden, P. A.; McDonald, I. R. Hydration and mobility of ions in solution. *The Journal of Physical Chemistry* **1983**, *87*, 5071–5083, DOI: 10.1021/j150643a008.

(S4) Young, J. M.; Panagiotopoulos, A. Z. System-Size Dependence of Electrolyte Activity Coefficients in Molecular Simulations. *The Journal of Physical Chemistry B* **2018**, *122*, 3330–3338, DOI: 10.1021/acs.jpcb.7b09861.
